# The role of the right to life in respect of deaths caused by negligence in the healthcare context

**DOI:** 10.1093/medlaw/fwad037

**Published:** 2023-11-25

**Authors:** Elizabeth Wicks

**Affiliations:** Centre for Rights and Equality in Health Law (CREHL) and Leicester Law School, University of Leicester, Leicester, UK

**Keywords:** Article 2 ECHR, Human Rights, Medical negligence, Negligent deaths, Positive obligations, Right to life

## Abstract

This article investigates the question of whether a death caused by negligence in the healthcare context is capable of violating the right to life under Article 2 of the European Convention on Human Rights. This provision imposes extensive positive obligations upon Contracting States, including an operational duty to take reasonable steps to save a life that they know, or ought to know, is at risk. This article addresses the question of exactly when such an operational duty arises, with particular focus on the healthcare context in which deaths caused by medical negligence have not traditionally been regarded as amounting to violations of the right to life. This article argues that two key factors in determining the existence of an operational duty to save life are the assumption of responsibility and nature of risk. It also argues for the need to take surrounding circumstances into account and for an increased use of the right to life in holding public bodies to account for deaths caused by negligence in the healthcare context.

## I. INTRODUCTION

A human rights approach to healthcare is now well-established,^1^ but one aspect of medical law often evades a human rights focus: medical negligence.^2^ Appropriate standards of care in diagnosis and treatment remain governed by traditional tort law standards which, under the (even refined) *Bolam* test, means responsible professional standards and thus continue to emphasise the duties of doctors rather than the rights of patients.^3^ One partial, but significant, reason for the gulf between medical negligence and human rights law is the legacy of the *Powell v United Kingdom* admissibility decision,^4^ in which the European Court of Human Rights (the Court) held that a death resulting from medical negligence does not generally constitute a breach of Article 2 of the European Convention on Human Rights’ (ECHR) right to life. More recently, the Grand Chamber judgment in *Lopes de Sousa Fernandes v Portugal*,^5^ reaffirmed this approach. It is a judgment that is vulnerable to the criticism that it seeks to limit the applicability of the right to life in the healthcare context as compared with other contexts, such as prisons or criminal justice. This article seeks to clarify the potential application of the right to life in the specific context of deaths caused by medical negligence. This is an increasingly significant issue given the unprecedented pressures faced by the National Health Service (NHS) in recent years. Reports of lengthy delays for ambulances, and in Accident and Emergency departments, have become more frequent and raised concerns about lives being put at risk.^6^ Furthermore, reports of substandard care being provided in certain hospitals and care homes also raise the possibility of patients dying due to a negligent standard of care.^7^ It is, therefore, a timely moment to seek clarity on the role of Article 2’s protection of the right to life in the context of negligently caused deaths within the public healthcare system. Following a general overview of the positive obligations to protect life under this provision, this article investigates state liability for negligent deaths in the healthcare context and critiques the current ambiguous position following *Lopes de Sousa Fernandes*. This article then seeks to identify the factors that should be relevant to determining when an operational duty arises under Article 2, and argues that both an assumption of responsibility and the nature of risk are crucial factors. Finally, due to Article 2’s underlying principle of respect for all human life, it is argued that in assessing the reasonableness of a state’s actions, all of the surrounding circumstances leading to the death, including where appropriate issues of inadequate funding, planning or policy, should be given significant weight.

## II. POSITIVE OBLIGATIONS TO PROTECT LIFE UNDER ARTICLE 2 ECHR

The right to life is often described as the most fundamental of all human rights. Without one’s life being secured, other rights will be of limited utility. It is not, however, an absolute right in any of its manifestations^8^ and its ambit and scope remain ambiguous and controversial.^9^ This article will focus on the right to life as protected in Article 2 of the ECHR. This provision is binding on the United Kingdom (UK) as a matter of international law due to the UK’s ratification of this regional treaty in the early 1950s. It has also, of course, been incorporated into the domestic law of the UK in the Human Rights Act 1998 (HRA). Due to this statute, Article 2 is binding on all public authorities, who will act unlawfully if they violate the right to life.^10^

At its core, Article 2 prohibits the intentional deprivation of life, although there are exceptions to this prohibition in certain circumstances.^11^ Of greater relevance to the healthcare context are the positive obligations inherent in the right to life. They stem from the opening phrase of Article 2, which states that ‘Everyone’s right to life shall be protected by law’. This protection requires far more than a negative obligation on the state not to kill; it also requires positive steps to be taken to preserve human life. The question posed by this article is whether a death caused by the negligence of a public authority, particularly in cases where the authority has failed to act in a way that could have prevented death, violates Article 2.

The starting point for this discussion is the landmark case of *Osman v United Kingdom*,^12^ for it is this case that sets out in the clearest terms the potential scope of the positive obligations required under Article 2 within the context of the failure of a public body. In this case the police, to take steps that may have saved a human life. In *Osman*, the Court outlined the positive obligation imposed upon a state party under Article 2, explaining that this provision ‘enjoins the state not only to refrain from the intentional and unlawful taking of life but also to take appropriate steps to safeguard the lives of those within its jurisdiction’.^13^ The Court further explained that there are two levels to the positive obligation to safeguard life. The first of these is the primary duty to provide effective criminal law provisions, backed up by law-enforcement machinery. The state is always under this duty, and as we will see, there are comparable duties in relation to healthcare. At the second level, the positive obligation may also extend to the need for *preventive* operational measures to protect an individual whose life is at risk from the criminal acts of another individual. In respect of this, the Court established the important principle that state authorities must do all that could reasonably be expected of them to avoid a real and immediate risk to life of which they have, or ought to have, knowledge.^14^ However, the Court recognised that this obligation must be a limited one due to ‘difficulties involved in policing modern societies, the unpredictability of human conduct and the operational choices which must be made in terms of priorities and resources’.^15^ The obligation must, therefore, be interpreted in a way which does not impose an impossible or disproportionate burden on the authorities.^16^ The Court in *Osman* also held that the positive obligation of the police to safeguard lives is subject to the requirements of due process, including those implied by Article 5 of the ECHR’s right to liberty and Article 8 of the ECHR’s right to respect for private life. This recognition of the need to balance not only public resources but also potentially conflicting Convention rights, is one of the most interesting elements of the positive obligations imposed by the right to life. It means that while the state sometimes has a duty to act to save life, that duty can be outweighed by duties under other rights, such as the obligations not to interfere with a person’s private life or autonomy or not to impose degrading treatment. In the case of *Osman*, the relevant conflicting rights were those of the suspect, but often the conflicting rights can be those of the very person whose life is at risk. For example, if the threat to life comes from suicide, the state has to balance a right to autonomy with a duty to save life. There is no easy way to achieve this and the Court has so far failed to provide clear guidance on such a dilemma.^17^

No violation of Article 2 was found in *Osman*, but many subsequent cases have found violations of the right to life in the context of public bodies failing to intervene to save life. For example, in *Opuz v Turkey*,^18^ the Court found Turkey to have failed in its positive obligation to protect the right to life of the applicant’s mother who had been killed in a domestic violence incident. She had told the authorities that her life was in immediate danger but their only response was to take statements from the perpetuator about the allegations against him. Similarly, in *Branko Tomašić and others v Croatia*,^19^ the Court held Croatia responsible under Article 2 when a death occurred after the premature release of a person from detention without a proper assessment of risk. In this case, the perpetuator had been found guilty of repeatedly threatening to kill himself and his family and was released after five months’ imprisonment without any adequate psychiatric treatment being provided to him and with no assessment of whether there was still a risk he would carry out his previous threats. It is clear from such cases that there is an operational duty imposed in some circumstances under Article 2 and that states can be found in violation of the right if they fail to take reasonable steps to save a life that they know, or ought to know, is at risk. However, the Court has traditionally been more reluctant to find a violation of Article 2 when a death occurs in the healthcare, as opposed to criminal justice, context. The vital question which will now be addressed is: when does such an operational duty arise in the healthcare context?

## III. WHEN DOES AN OPERATIONAL DUTY ARISE UNDER ARTICLE 2 IN THE HEALTHCARE CONTEXT?

In the healthcare context, Article 2’s positive obligations require states to make regulations compelling hospitals to adopt appropriate measures for the protection of patients’ lives, and they also require an effective independent judicial system to be set up so that the cause of death of patients in the care of the medical profession can be determined and those responsible made accountable.^20^ The distinction between procedural and substantive obligations under Article 2 is particularly prominent in the healthcare context, as procedural obligations to investigate deaths are often emphasised and findings of failures in process are, not surprisingly, viewed as less controversial than substantive failings leading to death. Criminal liability is rarely required for a death caused by medical negligence. Thus, if the infringement of the right to life is not caused intentionally, but rather negligently, it suffices if the domestic legal system affords victims a remedy in the civil courts.^21^ Within the UK, while there may be domestic criminal liability for extreme or ‘gross’ instances of negligence causing the death of the patient,^22^ and the possibility of a corporate manslaughter prosecution of an NHS Trust exists,^23^ there has not traditionally been any human rights liability for the hospital trust under the HRA 1998 nor the state itself under the ECHR. Given revelations about negligence and mistreatment within some UK hospitals in recent years, the exclusion of Article 2 from this arena has wide-reaching implications.^24^

In the important case of *Powell v United Kingdom*,^25^ the Court held that a death resulting from medical negligence does not generally constitute a breach of Article 2. In this case, the applicants’ 10-year-old son had died as a result of the negligence of his general practitioners and a lack of coordination between the various doctors treating him. In addition, the applicants alleged a cover-up after their son’s death and falsification of his medical records. The Court found their claim under Article 2 to be inadmissible because it was held that neither an error of judgement on the part of a health professional, nor negligent coordination among health professionals in the treatment of a particular patient, is sufficient to constitute a breach of the state’s positive obligations under this Article. It may also have been relevant to the dismissal of the application that the Powells had settled the medical negligence action out of court. Although *Powell* is only an admissibility decision, the Court’s approach has become influential and has limited the extent to which Article 2 has been applied in the healthcare context.

In recent years, however, there has been a retreat from the *Powell* position by the Court. Thus, in *Mehmet Şentürk and Bekir Şentürk v Turkey*,^26^ the Court made clear that a death caused by medical negligence *can* amount to a violation of Article 2 in certain circumstances. The Court’s explanation for this departure from *Powell* remains somewhat ambiguous. It said that, in *Mehmet*, ‘the negligence attributable to that hospital’s medical staff went beyond a mere error or medical negligence, in so far as the doctors working there, in full awareness of the facts and in breach of their professional obligations, did not take all the emergency measures necessary to attempt to keep their patient alive’.^27^ The case concerned the death of a pregnant woman and the fetus owing to errors of judgement by health professionals, as well as a failure to provide treatment on account of her inability to pay the hospital fees in advance. In a crucial finding, the Court held that ‘the deceased woman, victim of a flagrant malfunctioning of the hospital departments, was deprived of the possibility of access to appropriate emergency care’.^28^ This finding led the Court to conclude that the State had failed in its obligation to protect her physical integrity and thus had violated the substantive limb of Article 2. It is significant, however, that this is a case not about negligent medical treatment, but negligent failure to provide emergency treatment. This distinction has proven to be central to the Court’s approach to this area. Thus, there are other examples in which a negligent failure to provide emergency treatment has been found in violation of Article 2. For example, *Asiye Genç v Turkey*^29^ involved the death of newborn baby after being refused admission to public hospitals. A lack of coordination between hospitals led to the child’s death in an ambulance. The Court found a substantive violation of Article 2 due to the fact that the child was denied access to appropriate emergency treatment. The failure to offer treatment was said to constitute a denial of medical care such as to put a person’s life in danger.^30^ As Nissen has argued, this acknowledgement of positive obligations regarding access to emergency healthcare not only protects the lives of the poorest in society, but also safeguards a working environment which facilitates the responsibilities of health professionals in medical emergencies.^31^

In another case involving a failure to provide adequate emergency treatment which led to death, *Elena Cojocaru v Romania*,^32^ the Concurring Opinion of Judge Sajó explicitly distinguishes between a denial of healthcare and medical negligence. He argues that an issue may arise under Article 2 where it is shown that the authorities of a Contracting State have put an individual’s life at risk by denying healthcare which they have undertaken to make available to the population in general.^33^ But, Judge Sajo argued, neither an error of judgement on the part of a healthcare professional nor negligent coordination among healthcare professionals in the treatment of a particular patient should amount to a state violation of Article 2. Such an interpretation would keep the *Powell* approach to medical negligence alive. However, the emphasis placed upon it by Judge Sajo in *Elena Cojocaru* stems from the fact that the majority of the Court did not appear to be sticking to the distinction he proposed.^34^ In contrast to Judge Sajó’s finding of only a procedural violation of Article 2, the majority founnd a substantive violation as well on the basis that the apparent lack of coordination of medical services and a delay in administering the appropriate emergency treatment attested to a dysfunctionality of the public hospital services.^35^

The Court seemed to further depart from the *Powell* approach in the Chamber judgment in the case of *Lopes de Sousa Fernandes v Portugal*.^36^ This case cannot be categorised as a failure to treat but it involves the negligent coordination of information between units of the same hospital. The applicant alleged a violation of the right to life on account of the death of her husband, who died following a series of medical problems that occurred after he had undergone minor surgery for the removal of nasal polyps. In particular, she claimed that her husband had suffered a hospital-acquired infection, which led to bacterial meningitis; that this was not initially diagnosed by the emergency department due to a lack of coordination between the ENT department and the emergency department; that this delay in diagnosis had allowed a life-threatening infection to develop, which had then had to be treated with very high doses of medication with damaging side effects; and that the decisions to discharge her husband were not accompanied by the requisite medical follow-up. In its Chamber judgment, the Court found a violation of Article 2 on the basis that the risk of meningitis ‘was sufficient to warrant immediate medical intervention in accordance with the medical protocol for post-operative supervision’ and that the lack of coordination between the different hospital departments ‘attests to failings in the public hospital service’, leading the Court to conclude that the applicant’s husband was ‘deprived of the possibility of access to appropriate emergency care’.^37^

It is difficult to reconcile this conclusion with *Powell* because, unlike the other cases mentioned above, this was not about a failure to provide emergency care but rather a negligent coordination of information between hospital departments. Hyam has cogently argued that:

[H]owever one describes the dysfunctional communication between the Emergency and ENT departments in relation to the diagnosis of meningitis, it was no more or less ‘negligent’ than the kind of miscommunication which is regularly seen between state bodies (or internal units of state bodies) regularly at inquests, or in clinical negligence trials and is consistently explained in those contexts as an individual error of judgment rather than a systemic failure and a violation of Article 2.^38^

The dissenting judges in the Chamber judgment expressed some concern about viewing medical negligence cases as substantive violations of Article 2, arguing that this would turn the Strasbourg Court into a first- and last-instance medical malpractice court.^39^ While the majority confirmed that it is not for the Court to question the clinical judgement of healthcare professionals, nonetheless the Court relied upon expert evidence suggesting that meningitis was a complication that could arise in exceptional cases following a polypectomy, and they did this despite the fact that the applicant’s claims alleging medical negligence were found to be unsubstantiated by all domestic courts and enquiries.^40^

It is worth noting that the role of causation seems to be relaxed in relation to violations of Article 2 for deaths caused by negligence.^41^ In *Fernandes*, there was no obvious link between the delay in the diagnosis of meningitis and the death that occurred four months later and was not directly caused by the meningitis. The Chamber merely stated that it did not wish to speculate on the deceased’s prospects of survival if his meningitis had been diagnosed earlier,^42^ but instead was focused on the broader failings of the public hospital service. One justification for this relaxed approach to causation is that causing death is never specifically required under Article 2. Rather, the provision focuses on whether the applicant is a victim of conduct which puts their life at risk, even if they subsequently survive. It may be, therefore, that the actual death in this case is irrelevant to the rights violation. Indeed, it has been established that the Court is willing to find a violation of the right to life in circumstances where there has been no loss of life at all,^43^ suggesting that the crucial consideration under Article 2 is ensuring the necessary respect for all human life. The recognition of the need to protect everyone from actions that put their life at risk, and thus fail to adequately respect it, means that establishing the cause of a particular death is not as crucial as under the domestic law of negligence.

Overall, the Chamber judgment in *Fernandes* is hard to reconcile with *Powell* and it suggested that the right to life was about to play a more significant and prominent role in the regulation of standards of healthcare in the Contracting States. However, the subsequent Grand Chamber’s judgment in *Fernandes* sought to restore the *Powell* principle to its previous dominant position. The Grand Chamber began by acknowledging the previous lack of clarity on the issue of Article 2’s applicability to negligent deaths in the healthcare context, noting that the present case provided an opportunity to ‘reaffirm and clarify the scope of the substantive positive obligations of States’ in cases concerning allegations of negligence occurring in the context of medical treatment in hospitals.^44^ It reaffirms the vital distinction between medical negligence in treatment and denial of health care. In doing so, it refers to a number of previous cases, including *Senturk*, *Asiye Genç*, *Cojocaru*, and *Aydoğdu*, noting that in all except *Cojocaru*, the Court had drawn a similar distinction between an arguable claim of a denial of immediate emergency care and cases which concerned allegations of mere medical negligence. Therefore, in the Grand Chamber’s view, the approach adopted in those cases ‘cannot be transposed to cases where the allegations concern mere medical negligence’.^45^ They are, instead, viewed as exceptional cases in which the fault attributable to the healthcare providers went beyond a mere error or medical negligence and concerned circumstances ‘where the medical staff, in breach of their professional obligations, failed to provide emergency medical treatment despite being fully aware that a person’s life would be put at risk if that treatment was not given’.^46^ The Court did not probe the specific reasons behind the failures to treat in these cases, other than to emphasise that they resulted from the dysfunctioning of hospital services due to deficiencies in the regulatory framework.^47^

In relation to cases of negligent treatment, the Court confirmed that its task can only be focused upon looking for deficiency in the regulatory framework of the state. Such deficiencies are rarely found by the Court (although there are a few exceptions)^48^. However, in relation to the denial of access to life-saving emergency treatment, the Court’s task is more complex. The Grand Chamber held that a State *can* be liable for the acts and omissions of healthcare providers in two specific (‘very exceptional’)^49^ circumstances. The first is where an individual patient’s life is knowingly put in danger by denial of access to life-saving emergency treatment (as was the situation in, for example, *Senturk*).^50^ The second circumstance is where a systemic or structural dysfunction in hospital services results in a patient being deprived of access to life-saving emergency treatment and the authorities knew about, or ought to have known about, that risk and failed to undertake the necessary measures to prevent that risk from materialising, thus putting patients’ lives, including the life of the particular patient concerned, in danger.^51^ This second circumstance is a clear application of the *Osman* test which applies in various circumstances.^52^ Cases such as *Aydoğdu* and *Asiye Genç*, which seemed to concern negligent coordination between different hospitals resulting in a denial of emergency care for newborn babies, were classified by the Grand Chamber as concerning a ‘structural issue linked to the deficiencies in the regulatory framework’,^53^ and thus fell within the second exceptional circumstance.

In order to identify the exceptional cases and to distinguish them from ‘mere negligence’, the Grand Chamber said that a number of factors, taken cumulatively, must be met: the acts and omissions of the healthcare providers must go beyond a mere error or medical negligence; the dysfunction at issue must be objectively and genuinely identifiable as systemic or structural in order to be attributable to the State authorities, and must not merely comprise individual instances where something may have been dysfunctional in the sense of going wrong or functioning badly; there must be a link between the dysfunction complained of and the harm which the patient sustained; and the dysfunction at issue must have resulted from the failure of the State to meet its obligation to provide a regulatory framework.^54^ In short, the Grand Chamber has declared that there must be systemic or structural dysfunction which is linked to harm and results from a lack of an adequately functioning regulatory framework. These requirements have been met with some criticism. Laavrysen has noted that, paradoxically, the Court seems to provide a lower level of protection than in cases of ‘mere’ medical negligence, because a failure to provide a regulatory framework would suffice in mere negligence cases to amount to a violation of Article 2, while for denial of emergency healthcare more is apparently required before Article 2 becomes applicable.^55^ This is, indeed, an odd development stemming from a judgment aimed at reducing the application of Article 2 to mere negligence cases.

In addition, the requirements do little to undermine the impression that healthcare is treated differently from other contexts in relation to the right to life. Judge Serghides, in his dissenting opinion, pointed out that the majority’s requirement that a denial of access to life-saving emergency treatment be ‘genuinely identifiable as systemic or structural in order’^56^ in order to be attributable to the State authorities, is not required in other contexts. He notes that

‘in no situation, other than healthcare situations, in which there is a serious risk threatening life and which triggers a substantive positive obligation on the part of the State to protect life, does the Court’s case-law require a systemic problem as a precondition for a possible violation of Article 2 of the Convention.’^57^

It is not clear why deaths by negligence in the healthcare context should be treated differently than deaths caused by the negligence of public authorities in other contexts, such as prison or criminal justice. While it might be argued that the police and prison service exercise authoritative powers, a more pertinent factor is the common assumption of responsibility (and/or control) in all contexts. The right to life in Article 2 imposes obligations upon States to take reasonable steps to preserve all human life within its jurisdiction. There is no obvious reason why that obligation should be reduced when it is clinicians rather than police officers whose actions or omissions have endangered life. Even the more tightly defined operational duty under Article 2 was described upon its creation in *Osman* as applying whenever state authorities know or ought to know about a risk to life. The factors relevant to the determination of when an operational duty arises will be considered in the section below, but it is argued here that the duty should apply within the healthcare context alongside others. The fundamental importance of the Convention’s protection for the right to life requires its stringent application in all circumstances where the value of human life is undermined by failures of the state.

The other dissenting judge in *Fernandes*, Judge Pinto De Albuquerque, suggested that the Convention rights have not yet been accorded the same respect within the healthcare context as within others:

In Europe, there was a time when the law did not enter prisons or army barracks, when wardens and officers were untouchable gods while prisoners and soldiers were insignificant subjects. That time is long over for prisons and army barracks. Regrettably, it is not yet over for hospitals. As the majority see it, the Convention should stay at the hospital door.^58^

In the UK, there has been a gradual move away from such an approach; a retreat from the ‘Bolamisation’^59^ of medical law and an increased recognition that patients are persons with rights and not merely recipients of duties of care.^60^ Even the Supreme Court acknowledged in its 2015 judgment in *Montgomery v Lanarkshire Health Board,* that the law regulating healthcare must focus on the rights of the patient, as capable and responsible adults, rather than adopt the more traditional paternalistic approach.^61^ This recognised the evolution of English medical law from an era of professional duties and deference to one of patient rights and involvement. The Convention rights, and particularly Article 8 ECHR, have been applied in a range of healthcare contexts, including reproduction and end of life.^62^ It would be a pity if the approach of the Grand Chamber in *Fernandes* denied the application of human rights in that most vital issue of the avoidable deaths of patients. Judge Pinto De Albuquerque claimed that the Court ‘has still to take practical steps to move issues of health care from useless rhetoric to human rights implementation’.^63^ In the context of a human rights system that places great emphasis upon the need to respect rights in practice as well as in theory,^64^ it is indeed important that the right to life has practical application within a context in which lives are often at risk. The counterargument to this is presented by the UK Government’s intervention in *Fernandes,* in which it argued that deficiencies in the provision of medical treatment by healthcare professionals and hospital staff should not engage the responsibility of the Contracting State under the substantive aspect of Article 2, but could only ever engage the procedural aspect of Article 2. The Government based this argument on the fact that the ECHR does not contain any express provision recognising a right to the provision of health care, nor a right to be provided with healthcare of any particular standard.^65^ While this is undeniably true, it does not mean that a core human right that is given stringent protection in the Convention, such as the right to life, should be excluded from the healthcare context. Such a position would undermine the right’s integrity and becomes increasingly difficult to sustain as the right is seen to be applied across disparate other contexts in which life is at stake.

Returning to the facts of *Fernandes*, the Grand Chamber found only a procedural and not a substantive violation of Article 2. It noted that the applicant’s husband had not been denied access to treatment and that, instead, the applicant’s complaint concerned whether the medical treatment provided to her husband had been deficient because of the negligence of the doctors who had treated him.^66^ Unlike the Chamber’s earlier judgment, the Grand Chamber did not regard the alleged lack of coordination between the two hospital departments as amounting to a dysfunction in hospital services capable of engaging the State’s responsibility under Article 2. It did not identify any evidence of a situation of structural or systemic dysfunctions in the healthcare services in question and thus no substantive issues were raised under Article 2. It concluded that ‘the examination of the circumstances leading to the death of the applicant’s husband and the alleged responsibility of the health professionals involved are matters which must be addressed from the angle of the adequacy of the mechanisms in place for shedding light on the course of those events’.^67^ Its finding of a procedural violation stemmed from both the domestic disciplinary and criminal proceedings being viewed as ineffective and the domestic compensation action failing to provide sufficient redress due to lengthy delay. In the context of procedural requirements, the Court emphasised an important general point that ‘where there is a prima facie arguable claim of a chain of events possibly triggered by an allegedly negligent act that may have contributed to the death of a patient … the authorities may be expected to conduct a thorough examination into the matter’.^68^ The Grand Chamber’s focus on a procedural, rather than substantive, violation in *Fernandes* is not surprising. In a range of areas in which the Court feels unable or unwilling to engage with the substantive requirements of a right, it falls back on procedural failings. This is particularly true in relation to other health contexts raised under Article 8, such as abortion and assisted dying.^69^ Its application of this approach under Article 2 in this context ensures that the focus remains on appropriate regulatory standards. Furthermore, it often appears that procedural and substantive issues are particularly difficult to distinguish under the right to life. Indeed, in the context of positive obligations, most cases in which a substantive violation is found also involve procedural failings, suggesting that when states fail to get the regulatory and/or investigatory process right, they may be found to have fallen short on substantive grounds too. This is no doubt because of the focus under Article 2 on ensuring adequate respect for all human lives; failing to set life-respecting standards or investigating unnecessary deaths may often fall short of the substantive requirements expected under Article 2.

The Court's struggles with applying Article 2 in the healthcare context highlight an ambiguity surrounding the circumstances in which an operational duty arises under this provision. The Grand Chamber’s judgment in *Fernandes* sought to restore some clarity to the issue and domestic courts have followed the Grand Chamber’s approach of seeking to draw and secure a distinction between deaths caused by mere negligence and deaths caused by a negligent failure to provide emergency treatment. For example, the UK case of *R. (on the application of Parkinson) v HM Senior Coroner for Kent* applied the *Fernandes* distinction, with the Divisional Court stating that

‘At the risk of over-simplification, the crucial distinction is between a case where there is reason to believe that there may have been a breach which is a “systemic failure”, in contrast to an “ordinary” case of medical negligence’.^70^

Indeed, according to one commentator the court in *Parkinson*

‘firmly slammed the door shut on arguing for “system failures” engaging Article 2 save in the most exceptional case of system “dysfunction” which denies a person emergency medical treatment despite being aware that the person’s life is at risk if the treatment is not given’.^71^

However, it is argued here that the Grand Chamber’s approach in *Fernandes* only further complicates an already difficult area. The facts of the *Fernandes* case did not involve a denial of treatment, nor were they indicative of ‘mere’ negligence such as an error of judgement made by a single doctor. Instead, the facts revolved around negligent coordination of different departments of the same hospital and a lengthy and complex list of alleged instances of negligence by different persons and departments. Real-life scenarios such as this are not easy to classify and remain in an ambiguous position following the Grand Chamber’s simplistic distinction between negligent treatment and denial of emergency treatment. A more coherent solution is needed in order to determine when an Article 2 operational duty exists within the healthcare context. Furthermore, the Grand Chamber’s insistence that where there is a denial of emergency life-saving treatment it must reveal a systemic or structural dysfunction, which is linked to harm and results from a lack of regulatory framework, in order to violate Article 2, is unduly demanding. It leaves the unfortunate impression that deaths caused by negligence in the healthcare arena are to be treated differently in terms of state liability under the right to life than deaths caused by negligence in the criminal justice context.^72^ The obligations imposed upon Contracting States under Article 2 require reasonable steps to preserve life across the entire range of state responsibility and thus such an implicit distinction is undesirable. Furthermore, given UK law’s historical reluctance to intervene in the healthcare context or to question medical discretion, it is particularly disappointing that there should be a perception of Convention rights being stopped at the hospital door, as alleged by Judge Pinto De Albuquerque. Therefore, this article will now seek to provide greater clarity on the circumstances in which an operational duty arises under Article 2, in order that its application to deaths caused by negligence in the healthcare context can be made more transparent.

## IV. RELEVANT FACTORS IN DETERMINING WHEN AN OPERATIONAL DUTY ARISES

### A. Assumption of responsibility

There are a number of factors that may be relevant to determining when a death caused by the negligence of a public body will be found to have violated Article 2. One factor that appears to be of great significance in determining when the operational duty to save life arises is that of an assumption of responsibility on the part of the state or a public body.^73^ Beyond the healthcare context, an assumption of responsibility is obviously apparent in cases concerning deaths in prison where individuals are directly under the control of the state. This might also be argued to apply to certain cases involving the police, such as the death of a witness,^74^ or after a 999 call,^75^ or where it is the presence of the police that has led to the risk to life. On this latter point, it is helpful to refer to the case of *Mikayil Mammadov v Azerbaijan,^76^* in which the applicant’s wife died by suicide by pouring a flammable liquid over herself and igniting it. She did this in response to a police operation conducted in a building in which she and her family were illegally residing. The Court concluded that the authorities could not be considered to have intentionally put the life of the applicant’s wife at risk, and that ‘self-immolation as a protest tactic does not constitute predictable or reasonable conduct in the context of eviction from an illegally occupied dwelling’.^77^ However, the Court proceeded to note that once the situation became clear, the police had an obligation under Article 2 to prevent the threat to life from materialising, by any means which were reasonable and feasible in the circumstances. This could be by, for example, trying to talk her out of any life-threatening actions or physically preventing her from pursing them, as well as providing immediate medical assistance. It is clear for the purposes of the present discussion that the mere presence on the scene of the police in this context had been a vital component in the risk to life, and thus specific duties to prevent the risk materialising could be imposed upon the police under Article 2. There are clear parallels with the UK case of *Robinson v Chief Constable of West Yorkshire Police*, in which a duty of care in negligence was finally recognised for the police but only in relation to dangers which the police themselves have created.^78^ Beyond such situations, it may be argued that the police cannot be regarded as having assumed responsibility for the lives of everyone in society and thus an operational duty would not be imposed in relation to other risks to life. This mirrors the conclusions of the Supreme Court in relation to the duty of care in *Robinson*, but it is here argued to also be applicable to the Article 2 operational duty. Thus, there needs to be some specific link between the person whose life is at risk and the police (such as a focused plea for help,^79^ or an ongoing relationship with the police),^80^ in order for an operational duty to arise.

In the healthcare context, UK courts under the HRA 1998 have been asked to grapple with the issue of the assumption of responsibility in the specific context of mental health patients. The Supreme Court first recognised a duty to prevent the suicide of compulsorily detained mental health patients in *Savage v South Essex NHS Trust*,^81^ and then subsequently in *Rabone & another v Pennine Care NHS Trust*^82^ extended that duty to voluntary mental health patients. This raises difficulties, however, because it encounters the *Powell* exclusion from the ambit of Article 2 of death by medical negligence. Lord Dyson in *Rabone* addressed this very point. He recognised that the Strasbourg Court had, in recent years, expanded the circumstances in which the operational duty is owed to include what may generally be described as ‘dangers for which in some way the state is responsible’.^83^ He referred to the *Mammadov* case, discussed above, as well as *Öneryildiz v Turkey*,^84^ which concerned an explosion at a large rubbish tip operated by the local authorities that caused a landslide engulfing part of a shanty-town located on the slope beneath the tip. Although the houses destroyed had been built illegally, the state authorities were still found to have violated Article 2 because they had tolerated the breach of planning controls and failed to install the ventilation ducts which they knew were required to make the tip safe. Thus, as Lord Dyson pointed out, this also fits in the ‘dangers for which the state is responsible’ category. He contrasted such cases with those involving hospital deaths resulting from what can be described as ‘casual acts of negligence’^85^ by medical staff. A patient undergoing major surgery may be facing a real and immediate risk of death, yet under the *Powell* principle there is no Article 2 operational duty to take reasonable steps to avoid the death of such a patient.^86^ Lord Dyson concluded that: ‘The operational duty will be held to exist where there has been an assumption of responsibility by the state for the individual’s welfare and safety (including by the exercise of control)'.^87^ He argued that an ‘exercise of control’ is not always required in order to engage the Article 2 operational duty. Lord Dyson claimed that in circumstances of sufficient vulnerability, the Strasbourg Court has been prepared to find a breach of the operational duty in the absence of an exercise of control by the state.^88^ Nonetheless, any underlying concern with vulnerability does not undermine the importance of the issue of the assumption of responsibility, which remains crucial to identifying when the operational duty exists.

### B. Nature of risk

The second factor relevant to an operational duty is the nature of the risk to life. This is recognised by Lord Dyson in *Rabone* when he distinguishes between an ‘ordinary’ risk of the kind that individuals in the relevant category should reasonably be expected to take and an ‘exceptional’ risk.^89^ There is also some support from the Strasbourg Court for this distinction. In *Stoyanovi v Bulgaria*,^90^ the Court rejected an application by the family of a soldier who died during a parachute exercise, drawing a distinction between risks which a soldier must expect as an incident of his ordinary military duties and ‘“dangerous” situations of specific threat to life which arise exceptionally from risks posed by violent, unlawful acts of others or man-made or natural hazards’.^91^ An operational obligation only arose in the latter situation. In this case, the Court noted that parachute training was ‘inherently dangerous but an ordinary part of military duties’ and therefore, in this context, the only substantive requirement on the State was to ensure ‘through a system of rules and through sufficient control that the risk is reduced to a reasonable minimum’. If the State does this, there will be no substantive liability under Article 2 even if the death is caused through the negligent conduct of an individual.^92^ Thus, it appears that where the risk to life is an ordinary risk of the kind to be expected by persons in a particular category (such as parachute training for soldiers, or perhaps violence from other prisoners), a death caused by the negligence of the relevant public body will not in itself violate Article 2 provided appropriate regulations are in place. Where the nature of the risk falls outside this ‘ordinary risk’ category however, the more stringent operational duty to take reasonable steps to prevent a specific risk to life will arise.

This explanation fits with the approach of the court in *R. (on the application of Maguire) v HM Senior Coroner for Blackpool and Fylde*,^93^ in which it was held that the operational duty was owed to vulnerable people under the care of the State for some but not all purposes. Specifically, the duty did not apply to the provision of ordinary medical treatment to a vulnerable person in a care home, in the absence of exceptional circumstances. More recently, the case of *R (Morahan) v West London Assistant Coroner*^94^ has also confirmed the relevance of the broader context and the nature of the risk to life. In this case, Popplewell LJ explicitly stated that:

the existence or otherwise of the operational duty is not to be analysed solely by reference to the relationship between the state and the individual, but also, and importantly, by reference to the type of harm of which the individual is foreseeably at real and immediate risk. This follows from the operational duty to protect life having the unifying feature of being one of state responsibility, and the need to focus on the scope of the duty which may be owed. There may be an operational duty to protect against some hazards but not others.^95^

Such a focus upon ‘state responsibility’ raises the inevitable question of exactly which risks are the responsibility of the state. Popplewell LJ concluded that control by the state cannot justify the imposition of the operational duty by reference to state responsibility ‘if the risk were of a type of harm which is unconnected to the control which the state has assumed over the individual’.^96^ This is a logical approach that ensures that the obligations imposed upon public bodies remain proportionate, and it also highlights the ongoing relevance of the issue of such bodies assuming responsibility for persons. Risks to life connected to that action are the type to which an individual (and likely a vulnerable individual) can legitimately expect the state to take reasonable steps to prevent.

It will also be important to consider the reasons for which a public body has assumed responsibility over an individual. In *Rabone*, the case concerning a voluntary mental health patient, it seems to be pertinent that the State only assumed responsibility for the patient because of the existence of a risk of suicide. The patient was admitted to the hospital because of concerns that she would try to take her own life and it is this factor that supported the application of an operational duty in this case, and the finding of a violation of Article 2 when, despite the initial hospital admission, the patient was not prevented from attempting suicide. Could it be that the reason underlying the State’s assumption of responsibility may be relevant to the establishment of an operational duty? In *Rabone*, the State assumed responsibility for this woman because she was at risk from suicide and then failed to protect her from that very risk. Similarly, in the *Branko Tomašić* case, mentioned above, the Strasbourg Court held Croatia responsible under Article 2 when a death occurred after the premature release of a person from detention without a proper assessment of risk.^97^ The State could be regarded as having assumed responsibility for the life of the deceased when it found the perpetuator guilty of repeatedly threatening to kill himself and his family, and imprisoned him for this reason. The failure to assess whether the risk to life still existed before release was vital to the finding of a violation in this case. It may be argued, therefore, that assumption of responsibility and nature of risk are connected and only after considering both factors can a conclusion be reached about the application of an operational duty.

Therefore, there are two questions that need to be asked when determining whether an operational duty arises under Article 2. First, has there been an assumption of responsibility by a public body? It is argued here that this question can be decided on the basis of certain categories of persons. For example, there will be an assumption of responsibility for all detainees and conscripted soldiers (as established by judgments of the Strasbourg Court) but also for non-conscripted soldiers,^98^ and, crucially for all patients, regardless of whether physical or mental health is impaired. Patients residing within a hospital are under the direct care of medical professionals. For tort law purposes, a duty of care is established.^99^ It is hard to regard this as anything less than an assumption of responsibility for the vulnerable individual. The second question that needs to be asked is whether the risk to life is of an exceptional nature or an ordinary risk for that category of protected persons? In terms of applying this approach to the healthcare context, I would argue that medical treatment carries with it inherent risks and that a risk to life from surgery is an ordinary risk for a patient. On this basis, even if there is an assumption of responsibility, the nature of the risk should not be regarded as one to which an Article 2 operational duty automatically attaches. This argument would explain, and substantiate, the *Powell* principle and do so on much clearer terms than the Grand Chamber’s *Fernandes* judgment. ‘Mere’ negligence tends to occur in relation to ordinary risks of medical treatment and thus does not engage the operational duty under Article 2. The emphasis in such cases, therefore, should remain focused on the regulatory level, despite the assumption of responsibility on the part of the hospital for the patient. A denial of emergency treatment, however, should not be categorised as such an ‘ordinary’ risk. Being denied basic treatment and care such that one’s life is endangered, for whatever reason, is not a risk that should be classified as an ‘ordinary’ risk to life within the healthcare systems of Contracting States of the ECHR. On this basis, it may be argued that in the context of a denial of emergency treatment an operational duty does arise. Of course, this does not necessarily mean that it will amount to a violation of Article 2, and the next section will consider the final piece of this puzzle: how to determine what steps are required to preserve a life obviously at risk.

### C. Assessing reasonableness: surrounding circumstances and respect for human life

It is important to note that even when the operational duty is engaged, it may still be difficult to establish a violation of the right to life. Negligence is, of course, easier to establish in many cases than a breach of Article 2, not least because the risk of death need only be reasonably foreseeable for negligence but must be both real and immediate for the operational duty under Article 2 to arise. In addition, proportionality and/or reasonableness are not concerns for negligence in relation to the breach of a duty of care, because, if a breach is established and causes harm, there is liability. But for human rights law, any interference with a right may be justifiable. Even the operational duty, after all, only requires *reasonable* steps be taken to avoid a risk to life materialising, and in recent cases in which Article 2 claims have been allowed to proceed, there has been a great emphasis placed upon the old adage from *Osman* that the positive obligation upon the state must be interpreted in a way which ‘does not impose an impossible or disproportionate burden on the authorities’.^100^

A broader look at the circumstances leading to the death may be helpful in determining the application of Article 2. For example, in relation to deaths in the criminal justice context under Article 2, the Strasbourg Court always places great emphasis upon the planning and control of the police operation that ultimately results in the deaths. In *McCann and others v United Kingdom*, the Court established (in its first opportunity to consider this Article) that Article 2’s protection for the right to life requires ‘most careful scrutiny’ not only of the actions of the state agents who administer lethal force but also of ‘all the surrounding circumstances including such matters as the planning and control of the actions under examination’.^101^ Even in circumstances where the deaths themselves are compatible with Article 2 (for example, because they are the result of an honest mistake, based upon good reasons, as to the necessity of the use of lethal force), States may be found in violation of Article 2 because the planning and organisation of the police operation had failed to minimise the risk to life.^102^ It is recognised by the Strasbourg Court’s approach to such cases that when an individual is killed by an armed officer of the state, it does not happen in isolation. The officer will have an extensive police operation supporting him and often that operation may be a lengthy one that observes the threat to life as it develops and potentially escalates. Therefore, there is more than one relevant choice in this context. The State must be held responsible not only for its agent’s last-minute decision to kill or not to kill, but also for the decisions that may have contributed to the need for such a choice. Indeed, the State often has the power to influence the development of the threat to life and, therefore, must do all that it reasonably can to prevent the threat developing to the stage where only the death of the suspect can repel it.

The same long-view approach can and should be taken in the context of health care. A link should be recognised between a final failure to take operational measures to save life and broader issues of respecting all human life through policies, planning, and operational oversight. The current crisis in the NHS highlights the importance of such contextual issues when considering individual stories of lives put at risk. These contextual issues may add substance to the assessment of reasonableness under Article 2’s positive obligations. Thus, a denial of emergency healthcare to a patient in need may be seen as simply the last in a long line of decisions that have resulted in the need for such a final life-threatening decision. The reasonableness of such a decision should not be assessed in isolation from those earlier choices. This argument has application in other contexts too, such as within prisons where a failure to prevent a suicide may or may not amount to a violation of Article 2, depending upon the surrounding circumstances. The Strasbourg Court can be seen to be heading in this direction in cases such as *Isenc v France*,^103^ where the violation of Article 2 was largely based upon the lack of evidence that a prisoner who died from suicide less than two weeks after being admitted to prison had been given a medical assessment of his mental state when arriving at the prison. Beyond the significance of a mental assessment, other relevant surrounding circumstances in a case such as this might include whether the prisoner’s choice to attempt suicide was an autonomous one owed some respect by the state, and also whether previous choices concerning prison conditions, available medical treatment, suitable cell mates and funding priorities, highlight a lack of respect for human life or not. In the healthcare context, as well as in others, such previous choices may reveal a more culpable state; a context in which there is something akin to a recklessness as to the preservation of human life, rather than a singular instance of negligence by a public body. And in such circumstances, Article 2 surely has a vital role to play.

## V. CONCLUSION

This article has investigated the role played by Article 2 of the ECHR’s protection of the right to life in the context of deaths caused by the negligence of public authorities in the healthcare context. It has focused upon the operational duty to take reasonable steps to save a life that the authorities know, or ought to know, is at risk. It has identified and critiqued a reluctance to apply this operational duty in the healthcare context, which was confirmed in the Grand Chamber judgment in *Fernandes*. Despite that Court’s expressed desire to enhance the clarity as to when an operational duty arises, it has been argued that the judgment does not provide a sufficiently clear or explicitly reasoned answer to this important question. Its simplistic distinction between negligent treatment and denial of emergency treatment is hard to apply in practice, and its requirements of systemic or structural dysfunction resulting from a lack of regulatory framework is unduly demanding. Therefore, this article has argued for the healthcare context to be treated in the same way as other contexts in relation to state liability under the right to life by focusing upon two crucial factors: an assumption of responsibility by a public body to be decided on the basis of certain categories of persons (with patients being argued here to fall under such an assumption) and the risk to life being of an exceptional nature rather than an ordinary risk for that category of protected persons. This still leaves room for interpretation based upon the facts of a case but at least provides a transparent framework for decisions in this area which is currently missing. Once it is established that an operational duty to take positive steps to save a life exists, the focus moves to a reasonableness assessment and it is argued here that all of the circumstances surrounding a decision whether or not to intervene must be taken into account, including where appropriate issues of inadequate funding, planning, or policy. This places the final life or death decision in the vital context of the state’s ongoing legal responsibility to respect all human life and cautions against a reckless disregard of the value of life. This obligation is of particular significance in the context of the inherently unequal relationships considered in this article between healthcare professionals and patients. Indeed, it is this unequal relationship which makes the application of human rights law particularly appropriate.^104^ Any reluctance to apply the right to life in these contexts should, therefore, be overcome. Article 2’s protection for the right to life is of fundamental importance within human societies that share an inherent respect for human life, and its full application within the healthcare context should be welcomed. The subtleties of Article 2 ensure that the obligations placed upon public bodies, and through them the State, will not be unreasonable ones, but the recognition of an obligation to take reasonable steps to save life should be used wherever appropriate in order to hold public bodies to account for negligent behaviour that threatens human life.

## Footnotes

1 Even the Supreme Court has now acknowledged that the law regulating healthcare must focus on the rights of the patient, as capable and responsible adults, rather than adopt the traditional paternalistic approach. (*Montgomery v Lanarkshire Health Board* [2015] UKSC 11.) See E Wicks, *Human Rights and Healthcare* (Hart 2007); M Grodin (ed), *Health and Human Rights in a Changing World* (Routledge 2013); T Murphy, *Health and Human Rights* (Hart 2014).

2 The term ‘clinical negligence’ is now more commonly used in this area of practice in England and Wales, but ‘medical negligence’ is adopted here as this is the term more frequently used within the Strasbourg Art 2 caselaw.

3
*Bolam v Friern Hospital Management Committee* [1957] 2 All ER 118; *Bolitho v City & Hackney Health Authority* [1998] AC 232; *McCulloch v Forth Valley Health Board* [2023] UKSC 26.

4
*Powell v United Kingdom* (App 45305/99), admissibility decision of 4 May 2000.

5
*Lopes de Sousa Fernandes v Portugal* (App 56080/13) 19 December 2017 [GC].

6 D Campbell, ‘Thousands Exposed to Severe Harm Due to Ambulance Wait Times Last Month’ *The Guardian* (London, 25 January 2023) <www.theguardian.com/society/2023/jan/25/record-exposure-to-severe-harm-from-england-ambulance-delays> accessed 17 February 2023.

7 See, for example, A Gregory, ‘Police Examine 600 Cases after Damning NHS Baby Deaths Report’ *The Guardian* (London, 20 March 2022) <www.theguardian.com/society/2022/mar/30/baby-deaths-inquiry-shrewsbury-nhs-trust-condemned-for-repeated-failures> accessed 17 February 2023, discussing the report on the failures of maternity care at the Shrewsbury and Telford Hospital NHS Trust.

8 Art 2 ECHR explicitly permits a deprivation of life resulting from the use of force which is no more than absolutely necessary: (i) in defence of any person from unlawful violence; (ii) in order to effect a lawful arrest or to prevent escape of a person lawfully detained; (iii) in action lawfully taken for the purpose of quelling a riot or insurrection. Art 6 ICCPR takes a similar approach through its use of the word ‘arbitrary’ in relation to prohibited deprivations of life. See, for example, *Suarez de Guerrero v Colombia* (HRC Communication 45/1979).

9 For example, questions continue to be asked about the position of the death penalty, abortion and assisted dying under the right to life. For greater detail, see E Wicks, *The Right to Life and Conflicting Interests* (Oxford University Press 2010).

10 The s 6(1) Human Rights Act 1998. Significantly, this includes the courts, but excludes the Westminster Parliament (s 6(3)).

11 Art 2(2) ECHR.

12
*Osman v United Kingdom* (App 23452/94) 28 October 1998.

13 ibid para 115.

14 ibid para 116.

15 ibid.

16 ibid.

17 See, for example, some of the prison suicide cases, where the focus is squarely on the question of whether the prison authorities ought to known of the suicide risk and the concept of autonomy (and indeed mental capacity) are rarely mentioned: *Keenan v United Kingdom* (App 27229/95) 3 April 2001; *Renolde v France* (App 5608/05, 16 October 2008); *Ketreb v France* (App 38447/09) 19 July 2012; *Çoşelav v Turkey* (App 1413/07, 9 October 2012); *Isenc v France* (App 58828/13) 4 February 2016); *Jeanty v Belgium* (App 82284/17) 31 March 2020). See also more recent cases adopting a similar approach in respect of suicide in mental health hospitals: *Hiller v Austria* (App 1967/14) 22 November 2016); *Fernandes de Oliveira v Portugal* (App 78103/14) 31 January 2019) [GC]. For detailed discussion of these issues, see E Wicks, *Suicide and the Law* (Hart 2023), Chapter 5.

18 App 33401/02, 9 June 2009.

19 App 46598/06, 15 January 2009.

20
*Vo v France* (App 53924/00) 8 July 2004 [GC], para 89, following *Powell v United Kingdom* (App 45305/99), admissibility decision of 4 May 2000, and *Calvelli and Ciglio v Italy* (App 32967/96) 17 January 2002, para 49.

21
*Calvelli and Ciglio*, ibid para 51, and quoted in *Vo*, ibid para 90.

22
*R v Adomako* [1995] 1 AC 171; *R v Misra and Srivastava* [2005] 1 Cr App R 21; *Rose v R* [2017] EWCA Crim 1168. The latter case held that in order to be potentially liable, there must be a serious risk of death that was, rather than ought to have been, foreseeable to the defendant, thus further limiting the potential application of this offence. For discussion see O Quick, ‘Medicine, Mistakes and Manslaughter: A Criminal Combination?’ (2010) 69 CLJ 18; M Brazier and N Allen, ‘Criminalising Medical Malpractice’ in CA Erin and S Ost (eds), *The Criminal Justice System and Health Care* (OUP 2007); and ‘Gross Negligence Manslaughter in Healthcare: The Report of a Rapid Policy Review’, led by Sir Norman Williams (2018) <assets.publishing.service.gov.uk/government/uploads/system/uploads/attachment_data/file/717946/Williams_Report.pdf> accessed 17 February 2023.

23 In the first use of the Corporate Manslaughter and Corporate Homicide Act 2007 against an NHS Trust, Maidstone and Tonbridge Wells NHS Trust were charged with the corporate manslaughter of Frances Cappuccini who died following a caesarean section operation. In accepting the submission of no case to answer, Coulson J suggested that a one-off failure may not suffice and that systemic failings are likely to be more relevant to this offence (*R v Cornish and Maidstone & Tunbridge Wells NHS Trust* (2015) EWHC 2967, para 99).

24 See, for example, the inquiry into unnecessary deaths at Gosport War Memorial Hospital <www.theguardian.com/society/2018/jun/20/gosport-war-memorial-hospital-opioid-drugs-policy-inquiry> and also the Report of the Mid Staffordshire NHS Foundation Trust Public Inquiry 2013 <webarchive.nationalarchives.gov.uk/20150407084231/http://www.midstaffspublicinquiry.com/report>.

25 See n 4 above.

26 App 13423/09, 9 April 2013.

27 ibid para 104.

28 ibid para 97.

29 App 24109/07, 27 January 2015.

30
*Aydoğdu v Turkey* ((App 40448/06) 30 August 2016) is a very similar case in which the death of newborn baby denied access to adequate emergency care in a public hospital was found to be the result of negligence and structural failings and again violated Art 2.

31 A Nissen, ‘A Right to Access to Emergency Health Care: The European Court of Human Rights Pushes the Envelope’ (2017) 26 Med LR 693, 697. To support his argument, Nissen cites UN CESCR, ‘General Comment 14 on the Right to the Highest Attainable Standard of Health’ (11 August 2000) UN Doc E/C 12/2000/4, para 42 which recognises the responsibilities of individuals including health professionals, regarding the realisation of the international right to health.

32 (App 74114/12) 22 March 2016.

33
*Cyprus v Turkey* (App 25781/94) 10 May 2001, para 219.

34 Kapelanska-Pregowska claims that *Cojocaru* ‘blurred the scope of State obligations and the distinction between medical negligence, denial of healthcare, and regulatory/systemic deficiencies’. She argues that this was a medical negligence case and did not reveal any regulatory or systemic deficiencies that could be attributable to the State. (J Kapelanska-Pregowska, ‘Medical Negligence, Systemic Deficiency, or Denial of Emergency Healthcare? Reflections on the European Court of Human Rights Grand Chamber judgment *in Lopes de Sousa Fernandes v Portugal* of 19 December 2017 and previous case law’ (2019) 26 EJHL 26, at 34.)

35
*Elena Cojocaru v Romania*, para 111.

36 App 56080/13, 15 December 2015.

37 ibid para 114.

38 J Hyam, ‘Mere Negligence May Breach Art 2 in NHS Hospital Cases’ (12 January 2016) <ukhumanrightsblog.com/2016/01/12/jeremy-hyam-mere-negligence-may-breach-art-2-in-nhs-hospital-cases/#more-27883> accessed 17 February 2023.

39 Chamber Judgment, joint dissenting opinion of Judges Sajó and Tsotsoria.

40 Chamber judgment, para 109.

41 On causation in human rights law, see G Turton, ‘Causation and Risk in Negligence and Human Rights Law’ (2020) 79 CLJ 148 and V Stoyanova, ‘Causation between State Omission and Harm within the Framework of Positive Obligations under the European Convention on Human Rights’ (2018) 18 HRLR 309.

42 ibid. *Fernandes* is a 5-2 decision. The two dissenting judges (Judges Sajó and Tsotsoria) dissent largely on the basis that there is no evidence that the negligence caused the death. They say that they ‘cannot understand how an alleged organisational negligence that did not result in death can be construed as the basis of State responsibility for failing to protect life (which is replaced with the term “physical integrity”)’.

43
*Makaratzis v Greece* (App 50385/99) 20 December 2004 [GC]. See *also Sašo Gorgiev v The Former Yugoslav Republic of Macedonia* (App 49382/06), 19 April 2012 and *Vilnes and Others v Norway* (App 52806/09 and 22703/10) 5 December 2013.

44 GC judgment (n 5) para 162.

45 ibid para 182.

46 ibid para 183.

47 ibid para 184.

48 See *Glass v United Kingdom* (App 61827/00) 9 March 2004 and *Arskaya v Ukraine* (App 45076/05) 5 December 2013.

49 GC judgment (n 5) para 190.

50 ibid para 191.

51 ibid para 192.

52 For example, violations of Art 2 were found in the following situations: *Öneryildiz v Turkey* (App 48939/99), 30 November 2004 [GC] (failure to ensure safety of shanty-town build illegally near a local authority rubbish tip); *Branko Tomašić and others v Croatia* (App 46598/06), 15 January 2009 (premature release of a person from prison without a proper assessment of risk); *Mikayil Mammadov v Azerbaijan* (App 4762/05) 17 December 2009 (failure by police to prevent suicide as a protest to eviction); and *Jasinskis v Latvia* (App 45744/08), 21 December 2010 (failure to provide medical attention to person in police custody).

53 GC judgment (n 5) para 184.

54 ibid para 194–96.

55 L Lavrysen, ‘Medical Negligence after *Lopes de Sousa Fernandes*: a Blank Check to the Member States with Respect to the Substance of the Right to Life?’ (<strasbourgobservers.com/2018/02/08/medical-negligence-after-lopes-de-sousa-fernandes-a-blank-check-to-the-member-states-with-respect-to-the-substance-of-the-right-to-life/#more-4090> accessed 17 February 2023).

56
*Fernandes* [GC] (n 5) para 195.

57 Dissenting opinion of Judge Serghides, para 12. He proceeds to argue that ‘One should not distinguish health-care situations from other situations which trigger the substantive positive obligation of a State to protect the lives of individuals, since the crux of the matter should be the protection of life and not the situation from which the risk to life arises’ (para 13).

58 Dissenting opinion of Judge Pinto De Albuquerque, para 93.

59 See M Brazier and J Miola, ‘Bye-Bye Bolam: A Medical Litigation Revolution?’ (2000) 8 Med LR 85. On the application of systemic negligence claims in the domestic context, see R Heywood (2021) ‘Systemic Negligence and NHS Hospitals: An Underutilised Argument’ (2021) 32 Kings L J 437.

60
*Chester v Afshar* [2004] 4 All ER 587; *Montgomery v Lanarkshire Health Board* [2015] UKSC 11.

61 ibid.

62 See, for example, *Evans v Amicus Healthcare Ltd* [2004] 3 All ER 1025. *R (Nicklinson) v Ministry of Justice; R (AM) v Director of Public Prosecutions* [2014] UKSC 38; *Re Northern Ireland Human Rights Commission’s Application for Judicial Review* [2018] UKSC 27; *Crowter and Others v Secretary of State for Health and Social Care* [2022] EWCA Civ 1559.

63 Dissenting opinion of Judge Pinto De Albuquerque, para 92.

64 For example, the ‘striking discordance between the theoretical right’ and the ‘reality of its practical implementation’ (*P and S v Poland* (App 57375/08), 30 October 2012, para 111) has been central to the Court’s findings of violations in many cases, including the abortion cases of *P and S v Poland* and *A, B and C v Ireland* (App 25579/05), 16 December 2010 [GC].

65 GC judgment (n 5) paras 157–58. The Government of Ireland also intervened along similar grounds.

66 ibid para 200.

67 ibid para 199.

68 ibid para 237.

69 See for example the cases on abortion under Art 8: *Tysiąc v Poland* (App 5410/03), 20 March 2007; *A, B and C v Ireland* (App 25579/05), 16 December 2010 [GC]; *RR v Poland*, (App 27617/04), 26 May 2011; *P and S v Poland* (App 57375/08), 30 October 2012.

70
*R (on the application of Parkinson) v HM Senior Coroner for Kent* [2018] EWHC 1501 (Admin), para 89.

71 J Hyam, ‘Slamming the Door on System Failure in Medical Negligence Inquests’ (19 June 2018) <ukhumanrightsblog.com/2018/06/19/slamming-the-door-on-system-failure-in-medical-negligence-inquests-jeremy-hyam-qc/> accessed 17 February 2023.

72 In the recent domestic case of *Maguire v HM Senior Coroner for Blackpool and Flyde* [2020] EWCA CIV 738, the Court of Appeal applied *Fernandes* and determined that the facts of the case before it were ‘remote from the sort of systemic regulatory failing which the Strasbourg Court has in mind as underpinning the very exceptional circumstances in which a breach of the operational duty to protect life might be found in a medical case’ (para 106).

73 There are links here of course with the issue of a duty of care in negligence, although they are separate concepts.

74 As in *Van Colle v Chief Constable of the Hertfordshire Police* [2008] UKHL 50.

75 As in *Michael and others v The Chief Constable of South Wales Police and another* [2015] UKSC 2.

76 (App 4762/05)17 December 2009.

77 ibid para 111.

78 [2018] UKSC 4.

79 Perhaps along the lines of the factual situation in *Michael* (n 75).

80 Such as in *Van Colle* (n 74).

81 [2008] UKHL 74.

82 [2012] UKSC 2.

83 ibid para 16.

84 (App 48939/99), 30 November 2004 [GC].

85 This phrase is used by Lord Rodger in *Savage* (n 81), para 45.

86
*Rabone* (n 82), para 21.

87 ibid para 22.

88 ibid para 23. Certainly the concept of vulnerability was subsequently given greater emphasis by the Strasbourg Court in *Fernandes de Oliveira v Portugal* (App 78103/14, 31 January 2019) [GC]. In this case, which involved the assessment of suicide risk in a mental health hospital, the Grand Chamber confirmed that authorities do have a general operational duty with respect to a voluntary psychiatric patient to take reasonable measures to protect him or her from a real and immediate risk of suicide, thus extending the duty to prevent suicide from detainees to voluntary patients. In reaching this conclusion, it explicitly relied upon the vulnerability of such patients (para 124).

89
*Rabone* (n 82) para 24.

90 App 42980/04, 9 November 2010.

91 ibid paras 59–61.

92 ibid para 61.

93
*R (on the application of Maguire) v HM Senior Coroner for Blackpool and Fylde* [2020] EWCA Civ 738.

94
*R (Morahan) v West London Assistant Coroner* [2021] EWHC 1603 (Admin).

95 ibid para 65.

96 ibid para 67.

97
*Branko Tomašić v Croatia* (n 52).

98 In respect of members of the armed forces, they are directly under the control of the state, with aspects of their autonomy suspended during service and acting directly on behalf of the state. The domestic case *of R (Smith) v Secretary of State for Defence* [2010] UKSC 29 supports the argument that members of the armed forces can in some circumstances be owed an operational duty.

99
*Barnett v Chelsea and Kensington Hospital Management Committee* [1969] 1 QB 428.

100
*Osman* (n 12), para 116, emphasised in *Smith* (n 98) para 81.

101 (App 18984/91) 27 September 1995, para 150.

102 For example, we may contrast *McCann* in which a violation of Art 2 was found in relation to the planning and control of an anti-terrorist operation that resulted in the death of three suspected terrorists, with *Andronicou and Constantinou v Cyprus* (App 25052/94) 9 October 1997) in which there was no violation despite both a hostage-taker and his victim being fatally shot by the police in an act of disproportionate force. Unlike in the earlier case of *McCann*, the planning and organisation of the Cypriot police operation was held to have minimised to the greatest extent possible the risk to life. The rescue operation as a whole, therefore, provided adequate protection for the right to life even though mistakes on the part of the officers involved led to two avoidable deaths.

103 App 58828/13, 4 February 2016.

104 See Chapter 1 in Wicks, *Human Rights and Healthcare*(n 1).

